# Neutrophil Extracellular Traps Exacerbate Secondary Injury *via* Promoting Neuroinflammation and Blood–Spinal Cord Barrier Disruption in Spinal Cord Injury

**DOI:** 10.3389/fimmu.2021.698249

**Published:** 2021-08-11

**Authors:** Zhou Feng, Lingxia Min, Liang Liang, Beike Chen, Hui Chen, Yi Zhou, Weiwei Deng, Hongliang Liu, Jingming Hou

**Affiliations:** ^1^Department of Rehabilitation, Southwest Hospital, Third Military Medical University (Army Medical University), Chongqing, China; ^2^Department of Neurosurgery, Southwest Hospital, Third Military Medical University (Army Medical University), Chongqing, China

**Keywords:** spinal cord injury, neutrophils, neutrophil extracellular traps, blood–spinal cord barrier, neuroinflammation

## Abstract

As the first inflammatory cell recruited to the site of spinal cord injury (SCI), neutrophils were reported to be detrimental to SCI. However, the precise mechanisms as to how neutrophils exacerbate SCI remain largely obscure. In the present study, we demonstrated that infiltrated neutrophils produce neutrophil extracellular traps (NETs), which subsequently promote neuroinflammation and blood–spinal cord barrier disruption to aggravate spinal cord edema and neuronal apoptosis following SCI in rats. Both inhibition of NETs formation by peptidylarginine deiminase 4 (PAD4) inhibitor and disruption of NETs by DNase 1 alleviate secondary damage, thus restraining scar formation and promoting functional recovery after SCI. Furthermore, we found that NETs exacerbate SCI partly *via* elevating transient receptor potential vanilloid type 4 (TRPV4) level in the injured spinal cord. Therefore, our results indicate that NETs might be a promising therapeutic target for SCI.

## Introduction

Spinal cord injury (SCI) is a devastating central nervous system (CNS) trauma due to its high mobility and tremendous social and financial burden (1). Unfortunately, current treatments for SCI are far from satisfactory (2), which should mainly be attributed to the limitations in understanding of its pathophysiological mechanisms (1). Generally, SCI consists of primary injury and subsequent secondary injury mechanisms (3). Primary injury refers to initial impact to the spinal cord caused by traumatic mechanical forces, while secondary injury is known as a series of biochemical, molecular, and cellular cascades that cause further damage (4). Since primary injury happens unexpectedly and cannot be prevented, targeting secondary injury mechanisms is crucial for SCI treatment (5).

As the first inflammatory cell recruited to the lesion site of SCI, neutrophils play significant roles in the secondary injury mechanisms of SCI (6). Neutrophils migrate to the injured spinal cord within hours and peak in 1 to 3 days after SCI (7, 8). After infiltrating, neutrophils produce and release pro-inflammatory mediators, oxidative enzymes (such as myeloperoxidase; MPO), proteolytic enzymes (such as matrix metalloproteinase-9 and elastase), and reactive oxygen species (ROS) to promote secondary damage, thus aggravating neurological deficit (7, 9, 10). In addition to secreting cytotoxic products, neutrophils were recently revealed to contribute to diverse diseases *via* releasing neutrophil extracellular traps (NETs) (11), an extracellular fibrous network firstly described by Brinkmann et al. (12). Except for CNS infections (13, 14), NETs were also demonstrated to be implicated in ischemic stroke (15), intracerebral hemorrhage (16), traumatic brain injury (17), and even neurodegenerative diseases (18). However, whether NETs contribute to pathophysiological changes in SCI remains unclear.

Therefore, we explored whether NETs promote secondary injury following SCI, and the potential mechanisms as to how NETs exacerbate SCI in the present study.

## Materials And Methods

### SCI Induction and Experimental Design

One hundred and ninety-eight female Sprague–Dawley (SD) rats (250–300 g; Army Medical University) were used in the present experimentation. All rats were maintained under a 12-h light/dark cycle condition with free access to food and water. Animal use protocols were approved by the Animal Care and Use Committee of the Army Medical University (NO. SYXK20170002).

The clip-compression SCI model was induced according to a previous method (19). Briefly, after anesthetized by pentobarbital (40 mg/kg; intraperitoneally), animals were subjected to T9 laminectomy using aseptic techniques. Then, a clip with 50-g closing force was used to compress the exposed spinal cord for 60 s to induce SCI. The same surgical procedure without compression was performed in sham-operated animals. Manual bladder emptying was carried out twice daily until the recovery of normal bladder control.

Animals were assigned to five groups: sham controls without spinal cord compression (sham group); SCI models with Cl-amidine (diluted in 5% DMSO; MedChemExpress; 50 mg/kg) treatment (Cl-amidine group); SCI models with vehicle (corresponding dose of 5% DMSO) treatment (DMSO group); SCI models with DNase1 (diluted in saline; Roche; 5 mg/kg) treatment (DNase1 group); and SCI models with vehicle (corresponding dose of saline) treatment (Saline group). For drug administration, Cl-amidine was administered through intraperitoneal injection after SCI induction, and DNase1 was administered through the tail vein 1 h after SCI induction. The dosage and timing of Cl-amidine and DNase I administration were performed based on the previous study (17).

### Immunofluorescence

Immunofluorescence labeling was performed as described previously (20). After being deeply anesthetized, animals were transcardially perfused with PBS and their spinal cords were removed. Obtained samples were fixed with 4% paraformaldehyde and then dehydrated with 30% sucrose solution. Serial longitudinal sections (10 μm) were prepared using a cryostat microtome. After washing in PBS containing 0.3% Triton X-100, sections were incubated with relevant primary antibodies at 4°C overnight. Then, sections were washed with PBS and incubated with appropriate secondary antibodies at 37°C for 2 h. Cell nuclei were stained using DAPI. Finally, stained sections were viewed and imaged under a confocal microscope (LSM-880; Zeiss). The following primary antibodies were used: rabbit anti-Myeloperoxidase (MPO, Abcam; 1:50), mouse anti-Histone H3 (citrulline R2+ R8 +R17) (Abcam; 1:100), mouse anti-glial fibrillary acidic protein (GFAP) (Biosensis; 1:500), rabbit anti-Laminin (Dako; 1:1000), mouse anti-CD31 (Abcam; 1:100), and rabbit anti-transient receptor potential vanilloid type 4 (TRPV4) (Abcam; 1:100).

SYTOX Orange staining was performed as previous described (21). Sections were stained with SYTOX Orange (Molecular Probes, Inc.) at a concentration of 5 μM for 10 min. Then, stained sections were viewed and imaged under a confocal microscope (LSM-880; Zeiss) after washing with PBS.

### Western Blot Analysis

Western blot (WB) was performed according to a previous method (22). Briefly, after animals were anesthetized and decapitated, spinal cord tissues of the lesion site were removed and collected immediately on ice (8). Then, samples were homogenized and lysed in RIPA buffer containing protease and phosphatase inhibitors. After centrifuging at 12,000 rpm for 10 min at 4°C, protein concentrations were determined using a BCA Assay Kit (Beyotime). Equal amounts of protein lysate (20 μg) were separated by 10% SDS-PAGE electrophoresis, followed by transferring onto PVDF membranes. After blocking in 5% fresh-non-fat skim milk prepared in TBST for 2 h at room temperature, membranes were incubated with the appropriate primary antibodies at 4°C overnight. Then, membranes were incubated with corresponding HRP-conjugated secondary antibodies for 2 h at room temperature after washing with TBST. Finally, protein bands were visualized with chemiluminescent HRP Substrate (Thermo Fisher) under Western Lightning-ECL (Bio-Rad, USA). The following primary antibodies were used: mouse anti-Histone H3 (citrulline R2+ R8 +R17) (Abcam; 1:1000), rabbit anti-ZO-1 (Abcam, 1:5000), rabbit anti-occludin (Abcam, 1:5000), rabbit anti-transient receptor potential vanilloid type 4 (TRPV4) (Abcam; 1:1000), and mouse anti-GAPDH (Zen-bio, 1:5000).

### Luminex Liquid Suspension Chip Assay

Luminex liquid suspension chip assay was applied to analyze inflammatory cytokines [including tumor necrosis factor (TNF)-α, interferon (IFN)-γ, interleukin (IL)-1β, IL-6, and IL-10], which was performed by Wayen Biotechnologies (Shanghai, China). Briefly, samples were obtained after spinal cord tissues from the same site of WB were lysed and centrifugated at 10,000 rpm for 10 min. After protein concentrations were measured, equal amount of protein (45 μg) sample was taken to diluted to equal volume (50 μl). Then, samples were incubated in 96-well plates embedded with microbeads for 1 h, after which they were incubated with detection antibodies for 30 min. Finally, streptavidin-PE was added into each well to be incubated for 10 min, and values were measured by the Bio-Plex MAGPIX System (Bio-Rad).

### TUNEL Staining

After washing in PBS containing 0.3% Triton X-100, sections were incubated with the primary antibody mouse anti-NeuN (Abcam; 1:200), which is used to mark neurons, at 4°C overnight and then incubated with appropriate secondary antibody at 37°C for 2 h. Subsequently, sections were incubated with TUNEL staining mixture (In Situ Cell Death Detection Kit, TMR red; Roche) at 37°C for 1 h. Cell nuclei were stained using DAPI. Finally, stained sections were imaged under a confocal microscope (LSM-880; Zeiss). TUNEL-positive cells and neurons were counted using ImageJ software (National Institutes of Health, USA).

### H&E Staining

Firstly, sections were stained with hematoxylin for 1 min and washed three times in distilled water. Then, sections were stained with eosin for 2 min. Stained sections were imaged using a light microscope.

### Electrophysiological Assessment

The functional integrity of spinal pathway was evaluated by motor evoked potentials (MEPs) according to a previous method (23). Briefly, after being anesthetized with 1% pentobarbital sodium (20 mg/kg; intraperitoneally), experimental animals were implanted with four monopolar needle electrodes in appropriate locations: one at the base of the nose (acting as the anode), one at the midpoint between two ears (acting as the cathode), one into the gastrocnemius muscle (recording electrode), and the last one at the base of the tail (ground electrode). The brain was excited by electrical pulse (intensity 10 mA; width 0.1 ms; rate 1 Hz), and the base-to-peak amplitude of MEPs was recorded.

### Behavioral Experiments

Motor function was evaluated with the Basso, Beattie, and Bresnahan (BBB) locomotor test on days 1, 7, 14, 21, and 28 after SCI induction. Briefly, experimental animals were observed by two evaluators to move freely in an open field for 5 min in a blinded manner. Motor function was evaluated according to the 0–21 BBB scoring. The average score of two evaluators was calculated to analysis.

### Blood–Spinal Cord Barrier Permeability Evaluation

Blood–spinal cord barrier (BSCB) permeability was determined using Evans blue (EB) dye extravasation as previously described with some modifications (24). Briefly, 24 h after SCI, 2% (w/v) EB dye (5 ml/kg, Sigma Aldrich) solution in saline was administered through the femoral vein. One hour later, rats were anesthetized and perfused with saline. For extravasation quantification, injured spinal cord was removed and weighed immediately. Then, samples were homogenized in 400 µl of 50% trichloroacetic acid and centrifuged at 10,000 *g* for 30 min. After incubating overnight at 4°C, samples were centrifuged at 10,000 *g* for 30 min, and supernatants were diluted fourfold with ethanol. Finally, fluorescence intensity was measured at 620/680 nm. Results were express as μg dye/g tissue.

For EB fluorescence, injured spinal cord was removed, fixed with 4% paraformaldehyde overnight, and dehydrated in 30% sucrose at 4°C. Then, samples were sectioned into 10-μm slices and observed using a confocal fluorescence microscope (LSM880, Zeiss).

### Statistical Analysis

Statistical analysis was performed using GraphPad Prism 8.0. Single comparison between two groups was analyzed by two-tailed Student’s *t* test. Multigroup comparisons were analyzed by one-way analysis of variance (ANOVA) followed by Bonferroni *post hoc* test. Data were presented as means ± standard deviation. *p* value below 0.05 was considered statistically significant.

## Results

### Infiltrated Neutrophils Produce NETs in the Injured Spinal Cord

**Figure 1:**
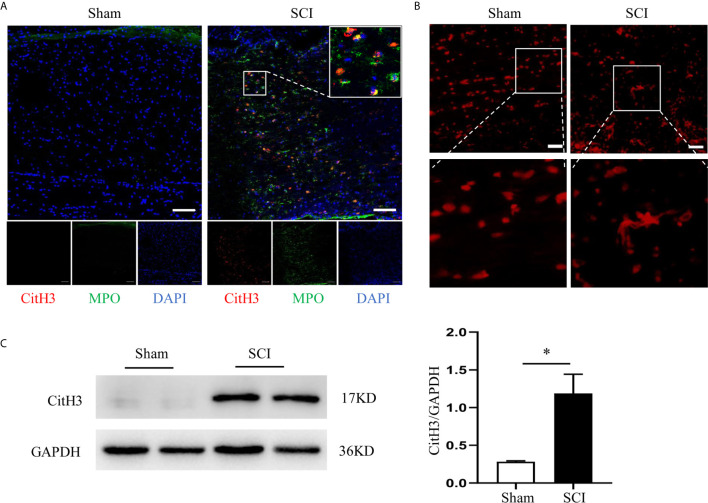
Infiltrated neutrophils produce NETs in the injured spinal cord. **(A)** Representative images of CitH3 (red) and MPO (green) double-positive cells in spinal cord from sham-operated rats and SCI rats at 24 h after operation. Nuclear was marked with DAPI (blue). Scale bars = 100 μm. **(B)** Representative images of network-like cell-free DNA structure by Sytox Orange staining. Scale bars = 200 μm. **(C)** Representative immunoblots and quantification of the CitH3 levels in spinal cord of rats subjected to SCI or sham operation. GAPDH is used as a loading control. Data are presented as means ± SD of *n* = 9 (*p < 0.05).

We first identified the presence of neutrophils and NETs at the epicenter of the SCI. Neutrophils peaked at 24 h and remained high until 3 days after SCI (**Supplementary Figure 1A**). At 24 h after SCI, a large number of neutrophils that were marked with MPO infiltrated into the injured spinal cord and produce NETs, which was characterized by citrullinated histone H3 (CitH3)^+^ neutrophils (**Figure 1A**). Furthermore, we visualized NETs (network of cell-free DNA structure) with SYTOX Orange staining in the lesion site at 24 h after SCI (**Figure 1B**). Quantification of the CitH3 levels at different time points in the injured spinal cord confirmed the presence of NETs (**Figure 1B** and **Supplementary Figure 1B**), while level of total histone H3 expression was not changed after SCI (**Supplementary Figure 2A**).

**Figure 2:**
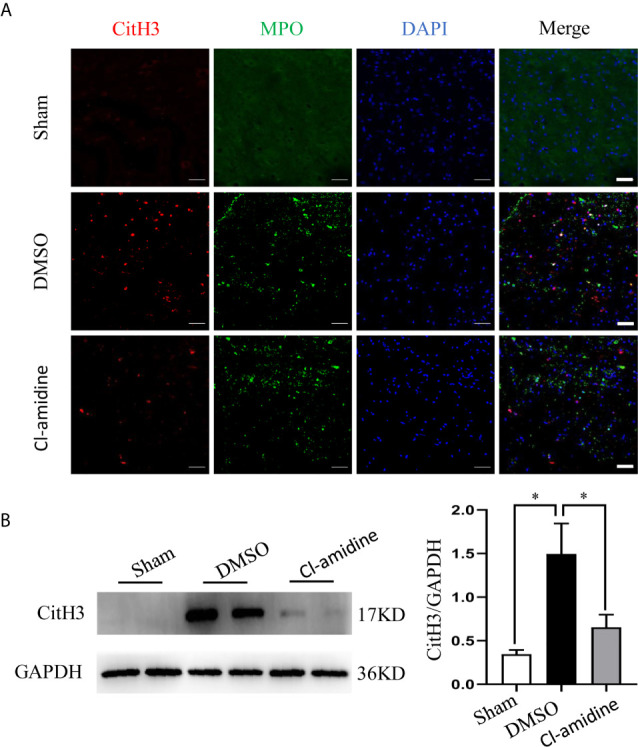
PAD4 inhibitor (Cl-amidine) prevents NETs formation after SCI. **(A)** Representative images of CitH3 (red) and MPO (green) double-positive cells in spinal cord from each group at 24 h after operation. Nuclear was marked with DAPI (blue). Scale bars = 50 μm. **(B)** Representative immunoblots and quantification of the CitH3 levels in spinal cord of each group. GAPDH is used as a loading control. Data are presented as means ± SD of *n* = 9 (*p < 0.05).

**Figure 3:**
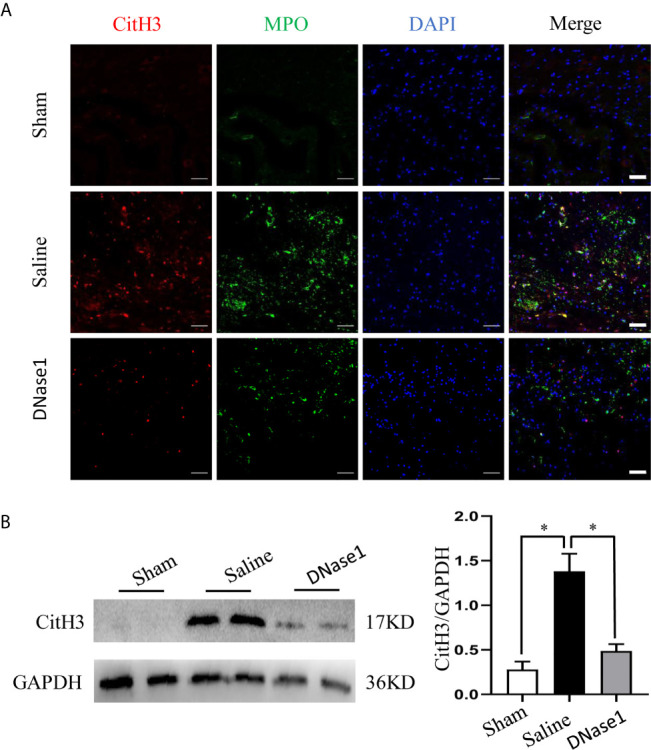
DNase1 degrades NETs after SCI. **(A)** Representative images of CitH3 (red) and MPO (green) double-positive cells in spinal cord from each group at 24 h after operation. Nuclear was marked with DAPI (blue). Scale bars = 50 μm. **(B)** Representative immunoblots and quantification of the CitH3 levels in spinal cord of each group. GAPDH is used as a loading control. Data are presented as means ± SD of *n* = 9 (*p < 0.05).

To demonstrate the effect of Cl-amidine, an inhibitor of enzyme peptidylarginine deiminase 4 (PAD4), which is the key enzyme mediating NETs formation, and DNase1 on restricting NETs, we firstly excluded the effect of vehicles (DMSO and saline) on NETs. We found that both DMSO and saline had no significant effect on the level of CitH3 in both sham and SCI rats (**Supplementary Figures S3A, B**). Administrating Cl-amidine reduced NETs significantly after SCI (**Figure 2**). Similarly, degrading NETs with DNase1 also significantly reduced the level of NETs in the lesion site (**Figure 3**). Both Cl-amidine and DNase1 administration had no significant effect on the level of total histone H3 expression in SCI rats (**Supplementary Figures S2B, C**). Although we found that the peak of NETs was observed at 3 days after SCI, early administration of both Cl-amidine and DNase1 also significantly reduced the NETs at later stage (**Supplementary Figure S4**). These results suggest that infiltrated neutrophils produce NETs at the epicenter after SCI, which were prevented by both Cl-amidine and DNase1.

### Restricting NETs Attenuates Neuroinflammation and Edema After SCI

**Figure 4:**
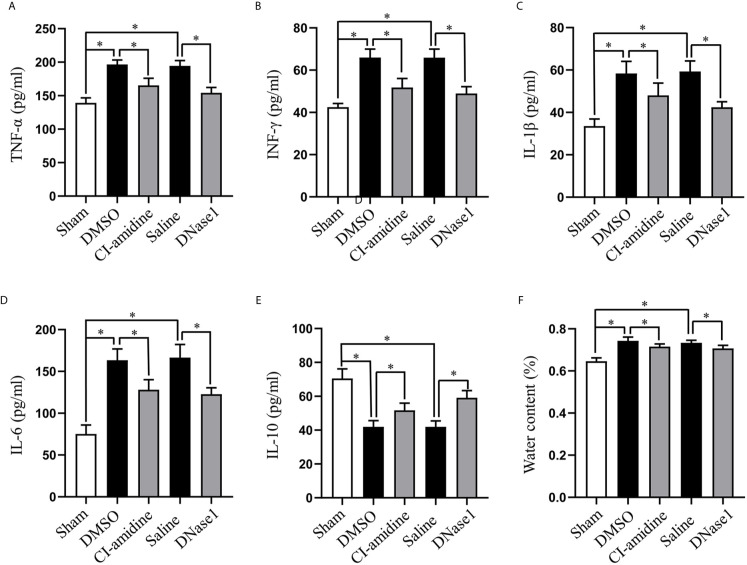
Restricting NETs attenuates neuroinflammation and edema after SCI. Levels of **(A)** TNF-α, **(B)** IFN-γ, **(C)** IL-1β, **(D)** IL-6, and **(E)** IL-10 in injured spinal cord of each group at 24 h after SCI. **(F)** Water contents of injured spinal cord from each group at 24 h after SCI. Data are presented as means ± SD of *n* = 6 (*p < 0.05).

Next, we evaluated the effect of NETs on neuroinflammation and edema after SCI. Local pro-inflammatory cytokines, including TNF-α, IFN-γ, IL-1β, and IL-6, were increased, while anti-inflammatory cytokine IL-10 was decreased at 24 h after SCI, which are all reversed by both Cl-amidine and DNase1 treatment (**Figures 4A–E**). Moreover, SCI-induced acute edema is also attenuated by Cl-amidine and DNase1 (**Figure 4F**). Thus, restricting NETs attenuated neuroinflammation and edema after SCI.

### Restricting NETs Reduces Cell Death After SCI

**Figure 5:**
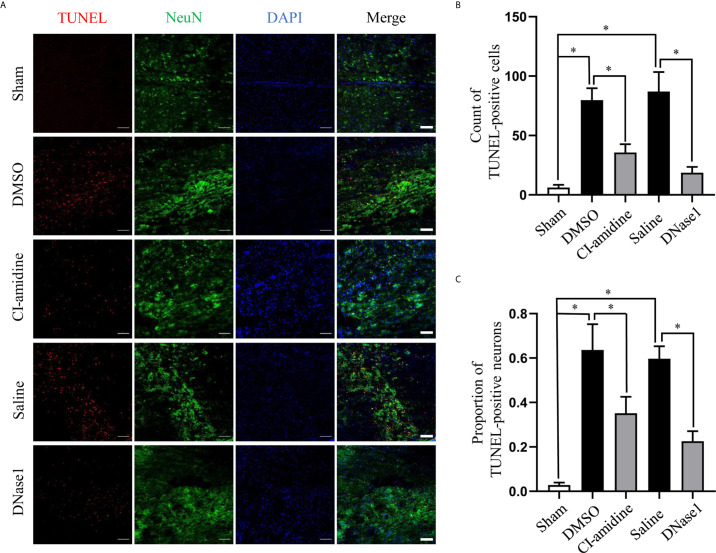
Restricting NETs reduces cell death after SCI. **(A)** Representative images of TUNEL (red) and NeuN (green) double-positive cells in injured spinal cord of each group at 24 h after operation. Nuclear was marked with DAPI (blue). Scale bars = 100 μm. Quantification of **(B)** TUNEL-positive cells and **(C)** double-positive cells in injured spinal cord of each group at 24 h after SCI. Data are presented as means ± SD of *n* = 6 (*p < 0.05).

Furthermore, we evaluated the effect of NETs on cell death after SCI. SCI induced massive cell death at the epicenter of lesion at 24 h post-SCI, which was suppressed by both Cl-amidine and DNase1 (**Figures 5A, B**). Furthermore, confocal images of co-staining of NeuN and TUNEL indicate that both Cl-amidine and DNase1 reduce neuron death after SCI (**Figures 5A, C**). This finding suggests that NETs is an important cause of cell death after SCI.

### Restricting NETs Reduces Scar Formation After SCI

**Figure 6:**
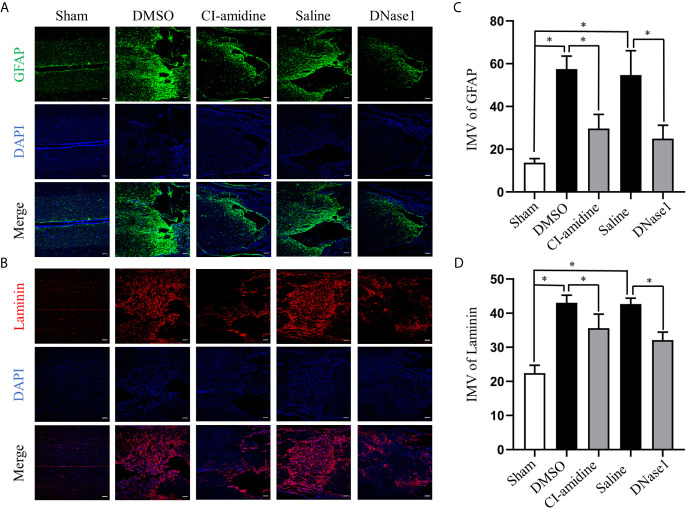
Restricting NETs reduces both glial and fibrotic scar formation after SCI. **(A)** Representative images of GFAP (green) positive glial scar in injured spinal cord of each group at 28 days after operation. Nuclear was marked with DAPI (blue). **(B)** Representative images of Laminin (red) positive fibrotic scar in injured spinal cord of each group at 28 days after operation. Nuclear was marked with DAPI (blue). Scale bars = 100 μm. Fluorescence intensity mean value (IMV) of **(C)** GFAP and **(D)** Laminin at the injury epicenter of each group. Data are presented as means ± SD of *n* = 6 (*p < 0.05).

To evaluate the effect of NETs on chronic phase of SCI, we assessed scarring (both glial and fibrotic) of lesion site at 28 days after SCI. Injury-induced glial scar formation, which is indicated by expression of GFAP, is significantly inhibited by both Cl-amidine and DNase1 administration (**Figures 6A, C**). In parallel, the amounts of laminin, which is the hallmark of the fibrotic scars hindering axon regeneration, in the lesions are significantly lower when NETs were restricted by Cl-amidine and DNase1 (**Figures 6B, D**). These results suggest that restricting NETs not only attenuates acute injury but also reduces chronic scar formation after SCI.

### Restricting NETs Reduces Tissue Damage and Promotes Motor Function Recovery After SCI

**Figure 7:**
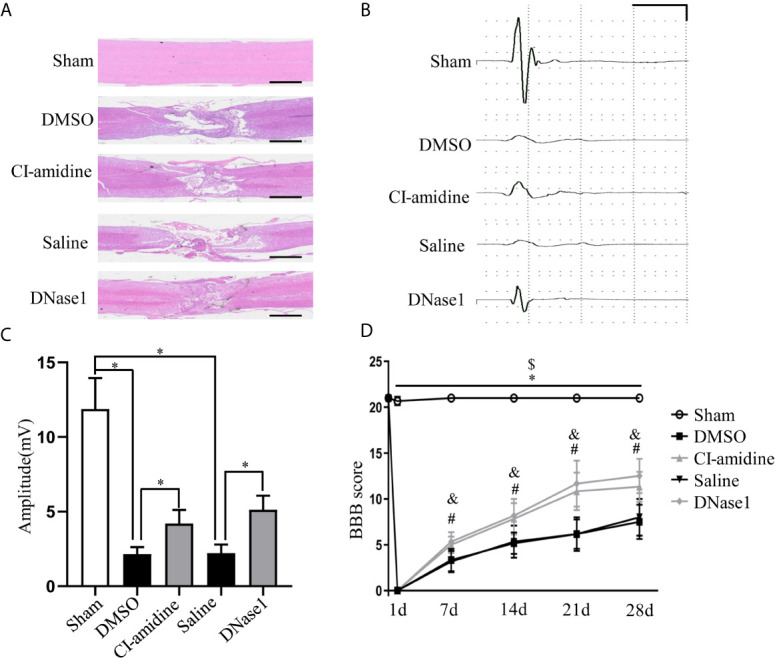
Restricting NETs reduces tissue damage and promotes motor function recovery after SCI. **(A)** Representative images of H&E staining performed on the injured spinal cord section of each group at 28 days after operation. Scale bars = 2 mm. **(B)** Representative recording of motor evoked potentials (MEPs) of each group at 28 days after operation. Scale: 5 mV/10 ms. **(C)** Amplitudes of MEPs of each group. Data are presented as means ± SD of *n* = 6 (*p < 0.05 *versus* vehicle). **(D)** The BBB scores at different time points of each group. Data are presented as means ± SD of *n* = 6 (*p < 0.05 sham *versus* DMSO, ^$^ *p* < 0.05 sham *versus* Saline, *p* < 0.05 CI-amidine versus DMSO, ^&^ *p* < 0.05 DNase1 *versus* Saline).

To evaluate the functional consequences of NETs restriction after SCI, we assessed functional integrity of spinal pathway by MEPs and motor function by BBB locomotor test. Both restricting NETs with Cl-amidine and DNase1 reduce tissue damage in the spinal cord at 28 days after SCI (**Figure 7A**). The representative records (**Figure 7B**) and amplitude (**Figure 7C**) of MEPs indicated that restricting NETs improve the functional integrity of motor pathway after SCI. Furthermore, motor function recovery evaluated by BBB test is significantly promoted following Cl-amidine and DNase1 treatment (**Figure 7D**). Taken together, these results suggest that restricting NETs reduced tissue damage and improved integrity of motor pathway and motor function recovery after SCI.

### NETs Promote BSCB Disruption, Which May Be Partly Through Elevating TRPV4

**Figure 8:**
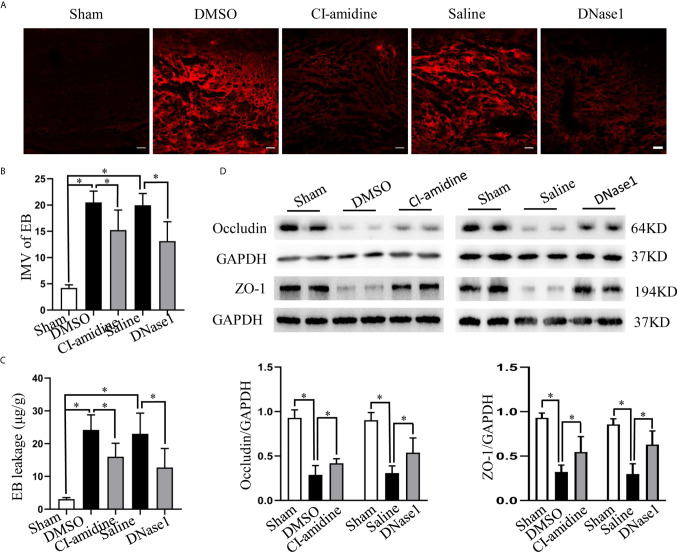
NETs promote BSCB disruption after SCI. **(A)** Representative fluorescence images of EB (red) leakage at the injury epicenter of each group at 24 h after operation. Scale bars = 100 μm. **(B)** Fluorescence intensity mean value (IMV) of EB at the injury epicenter of each group. **(C)** Quantification of EB leakage at the injury epicenter of each group at 24 h after operation. **(D)** Representative immunoblots and quantification of the BSCB tight junction proteins (ZO-1, occludin) levels in injured spinal cord of each group at 24 h after operation. Data are presented as means ± SD of *n* = 6 (*p < 0.05).

**Figure 9:**
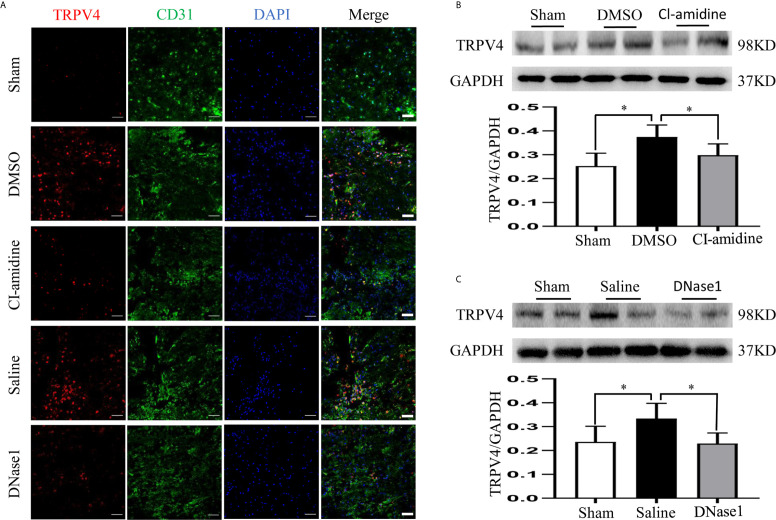
NETs promote BSCB disruption through elevating TRPV4. **(A)** Representative images of TRPV4 (red) and CD31 (green) double-positive cells at the injury epicenter of each group at 24 h after operation. Nuclear was marked with DAPI (blue). Scale bars = 50 μm. **(B, C)** Representative immunoblots and quantification of TRPV4 levels in injured spinal cord of each group at 24 h after operation. Data are presented as means ± SD of *n* = 6 (*p < 0.05).

Finally, we evaluated the effect of NETs on BSCB disruption and explored possible mechanisms. Both EB fluorescence and quantification of EB leakage indicate significant BSCB disruption at 24 h after SCI, which is ameliorated both by inhibiting NETs formation with Cl-amidine and degrading NETs with DNase1 (**Figures 8A–C**). In addition, the expression of tight junction proteins (ZO-1, occludin) that maintain the integrity of the BSCB is reduced after SCI, but prevented by Cl-amidine and DNase1 (**Figures 8D–F**). What is more, the expression of TRPV4 in CD-31-marked endothelial cells at the epicenter increases significantly after SCI, which is suppressed effectively by Cl-amidine and DNase1 (**Figures 9A–C**). Our results suggest that NETs promote BSCB disruption after SCI, which may be through, at least in part, elevating TRPV4.

## Discussion

In the present study, we have demonstrated that infiltrated neutrophils produce NETs in the lesion to exacerbate secondary injury *via* promoting neuroinflammation and blood–spinal cord barrier disruption after SCI. Both inhibiting NETs formation with Cl-amidine and degrading NETs with DNase1 reduce cell death, scar formation, and tissue damage, ultimately promoting motor function recovery, which benefits from alleviating neuroinflammation and blood–spinal cord barrier disruption partly by suppressing TRPV4.

In recent years, neutrophils were demonstrated to produce NETs in CNS under various pathological conditions to contribute to pathophysiology (11). In agreement with previous studies indicating the presence of NETs in stroke (15, 16) and traumatic brain injury (17), our present study demonstrated that infiltrated neutrophils produce NETs in the lesion after SCI.

NETs are generally decorated with granular and cytosolic proteins, proteases, and histones (12), which are induced by the activation of PAD4 (25). The latter converts arginine to citrulline on histones to promote chromatin decondensation (26); thus, it is essential for NETs formation (27). Despite the fact that neutrophil depletion is effective to block NETs formation, it is not an ideal treatment as the accompanying high risk of infection (17). Currently, inhibiting NETs formation with PAD4 inhibitor and degrading NETs with DNase1 are preferable strategies to restricting NETs (15, 17, 25). Our study corroborates the findings of previous researchers (15, 17), demonstrating that both Cl-amidine and DNase1 administration reduce NETs in CNS under pathological conditions.

Cell death, especially neuronal death, is a major pathological damage in the acute stage after SCI, which results from both primary and following secondary injury (4). Extremely preventing neuronal cell death is the goal of almost all neuroprotective therapies (2). Gratifyingly, we demonstrated that restricting NETs effectively reduces SCI-induced cell death, mainly neurons, in the present study. In addition, scar formation at the lesion site is also a common pathological response in SCI (28). Local scar tissue consists of two components: fibrotic scar contains extracellular matrix proteins (such as laminin, fibronectin, and collagen) in the lesion core, and glial scar contains reactive astrocytes surrounding the lesion core (28, 29). Glial scar has long been considered to be a barrier to inhibit axonal regeneration and a potential therapeutic target to facilitate neural repair (30, 31). In recent years, fibrotic scar receives growing attention in CNS diseases, especially SCI (32–34). Attenuation of fibrotic scar formation has been suggested to be a therapeutic target to facilitate neurological function recovery after SCI (35, 36). In our study described here, we found that both glial and fibrotic scars are reduced *via* restricting NETs, which facilitate motor function recovery ultimately after SCI. What is more, our results indicate that NETs formation might be a potential mechanism for how infiltrated immune cells drive CNS fibrosis (37).

As the main early pathophysiological changes following SCI, local neuroinflammation and BSCB disruption reinforce mutually and promote secondary injury after SCI (1, 2, 4). Preventing neuroinflammation and BSCB disruption are crucial strategies to block persistent secondary injury (38, 39). In the present study, we demonstrated that reducing NETs alleviates both neuroinflammation and BSCB disruption in injured spinal cord, hence ameliorating SCI and promoting functional recovery. Finally, we attempted to explore the potential mechanisms as to how NETs aggravate BSCB disruption and demonstrated that TRPV4 is upregulated in CD31-marked endothelial cells after SCI, which is suppressed by both Cl-amidine and DNase1. In agreement with our results, the nonselective cation channel TRPV4 was proved to contribute to endothelial and secondary damage after SCI, while inhibiting which attenuated SCI in a recent research (29). Combined with these results, we reasonably speculate that NETs aggravate BSCB disruption *via*, at least partly, elevating TRPV4 following SCI. Meanwhile, there are several limitations that require further studies in the present research. Firstly, *in vitro* study is needed to further confirm whether NETs damage endothelial cells through TRPV4 elevation. Secondly, whether NETs aggravate SCI through other mechanisms is worth investigating. Finally, all these results need to be verified in various models in the future.

**Figure 10:**
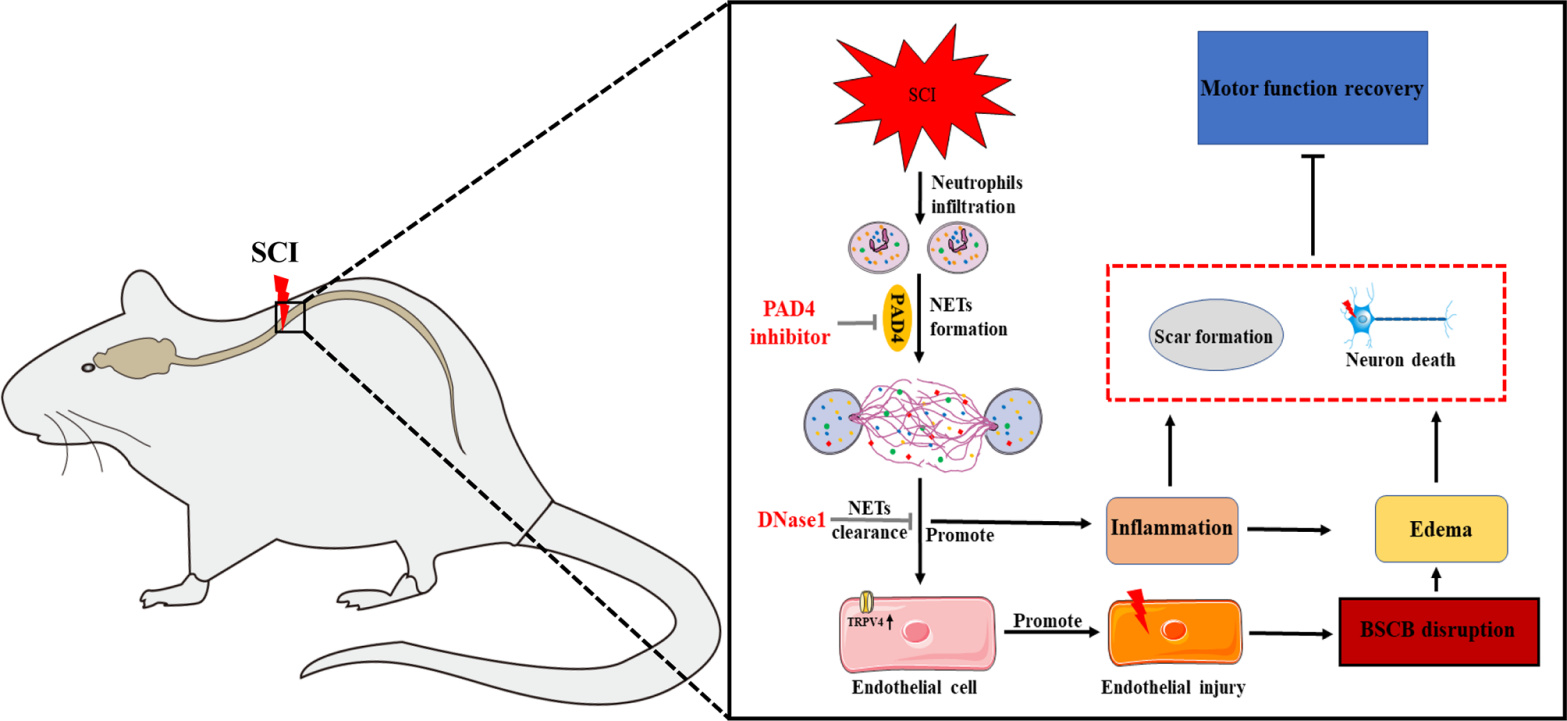
Mechanism of neutrophil extracellular traps (NETs) exacerbates secondary injury after spinal cord injury. Infiltrated neutrophils produce NETs, which subsequently promote neuroinflammation and blood–spinal cord barrier disruption to aggravate spinal cord edema and neuronal apoptosis partly *via* elevating transient receptor potential vanilloid type 4 (TRPV4) level following SCI in rats. Both inhibition of NETs formation by peptidylarginine deiminase 4 (PAD4) inhibitor and disruption of NETs by DNase 1 alleviate secondary damage and promote functional recovery after SCI.

In summary, our data demonstrated that NETs aggravate neuroinflammation and BSCB disruption, which may be partly *via* elevating TRPV4, to exacerbate secondary injury after SCI, while both inhibiting NETs formation and degrading NETs alleviate injury and promote motor function recovery (**Figure 10**). Therefore, our findings demonstrated that NETs may be a potential therapeutic target for SCI.

## Data Availability Statement

The original contributions presented in the study are included in the article/**Supplementary Material**. Further inquiries can be directed to the corresponding author.

## Ethics Statement

The animal study was reviewed and approved by Animal Care and Use Committee of the Army Medical University.

## Author Contributions

ZF, LM, LL, BC, HC, YZ, and WD contributed to the implementation of the experiment. HL and JH contributed to the design and paper writing. All authors contributed to the article and approved the submitted version.

## Funding

This work was supported by grants 81671211 (JH) and 81672251 (HL) from the National Natural Science Foundation of China and 2017MPRC-08 from the Talent Project of Southwest Hospital of China.

## Conflict of Interest

The authors declare that the research was conducted in the absence of any commercial or financial relationships that could be construed as a potential conflict of interest.

## Publisher’s Note

All claims expressed in this article are solely those of the authors and do not necessarily represent those of their affiliated organizations, or those of the publisher, the editors and the reviewers. Any product that may be evaluated in this article, or claim that may be made by its manufacturer, is not guaranteed or endorsed by the publisher.
