# Supplementary figures and images for: Neutrophil Extracellular Traps Exacerbate Secondary Injury via Promoting Neuroinflammation and Blood–Spinal Cord Barrier Disruption in Spinal Cord Injury
Source: Front Immunol. 2021 Aug 11;12:698249. doi: 10.3389/fimmu.2021.698249 (PMC8385494; doi:10.3389/fimmu.2021.698249)

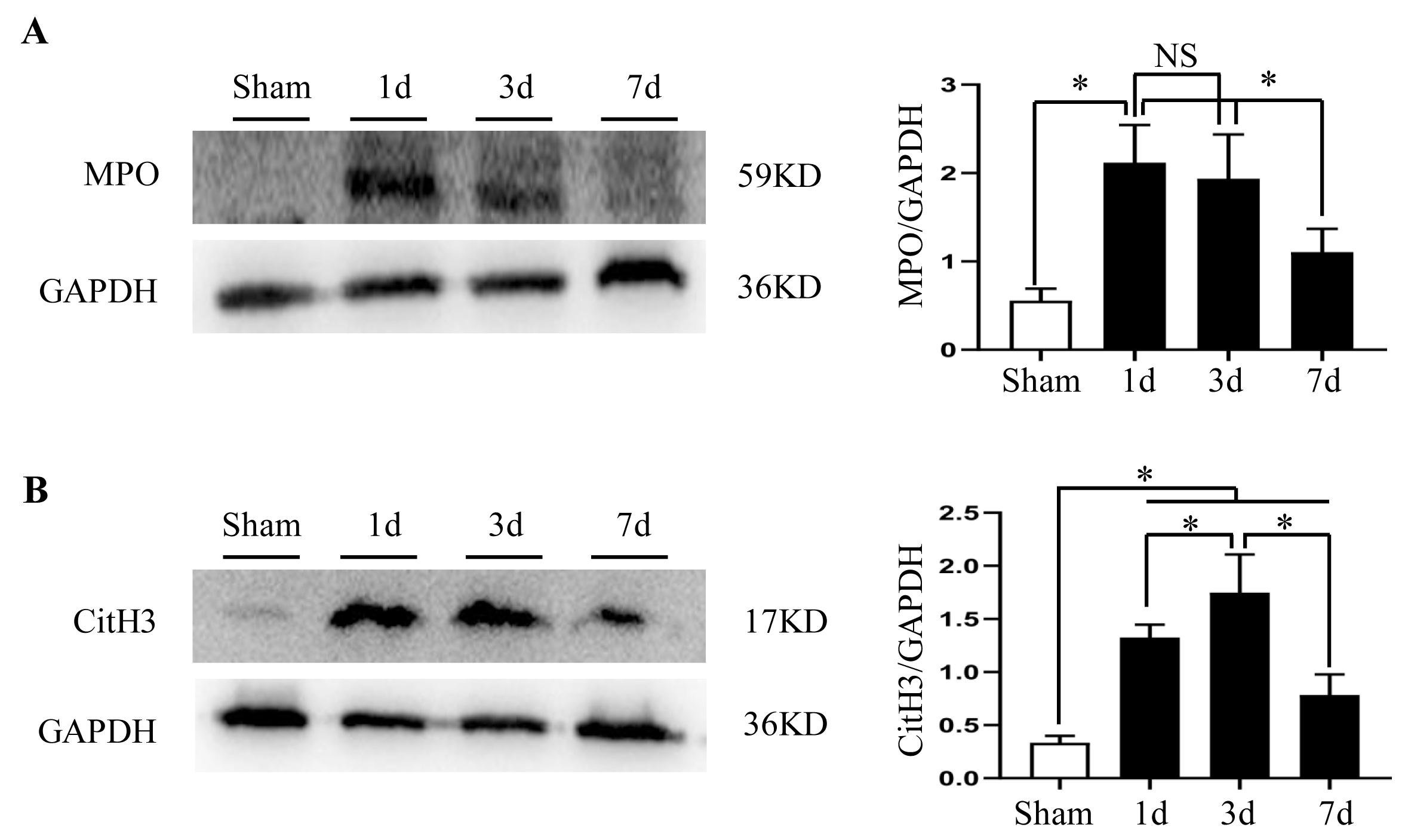

Supplement: Supplementary file 1 [file Image_1.tif]

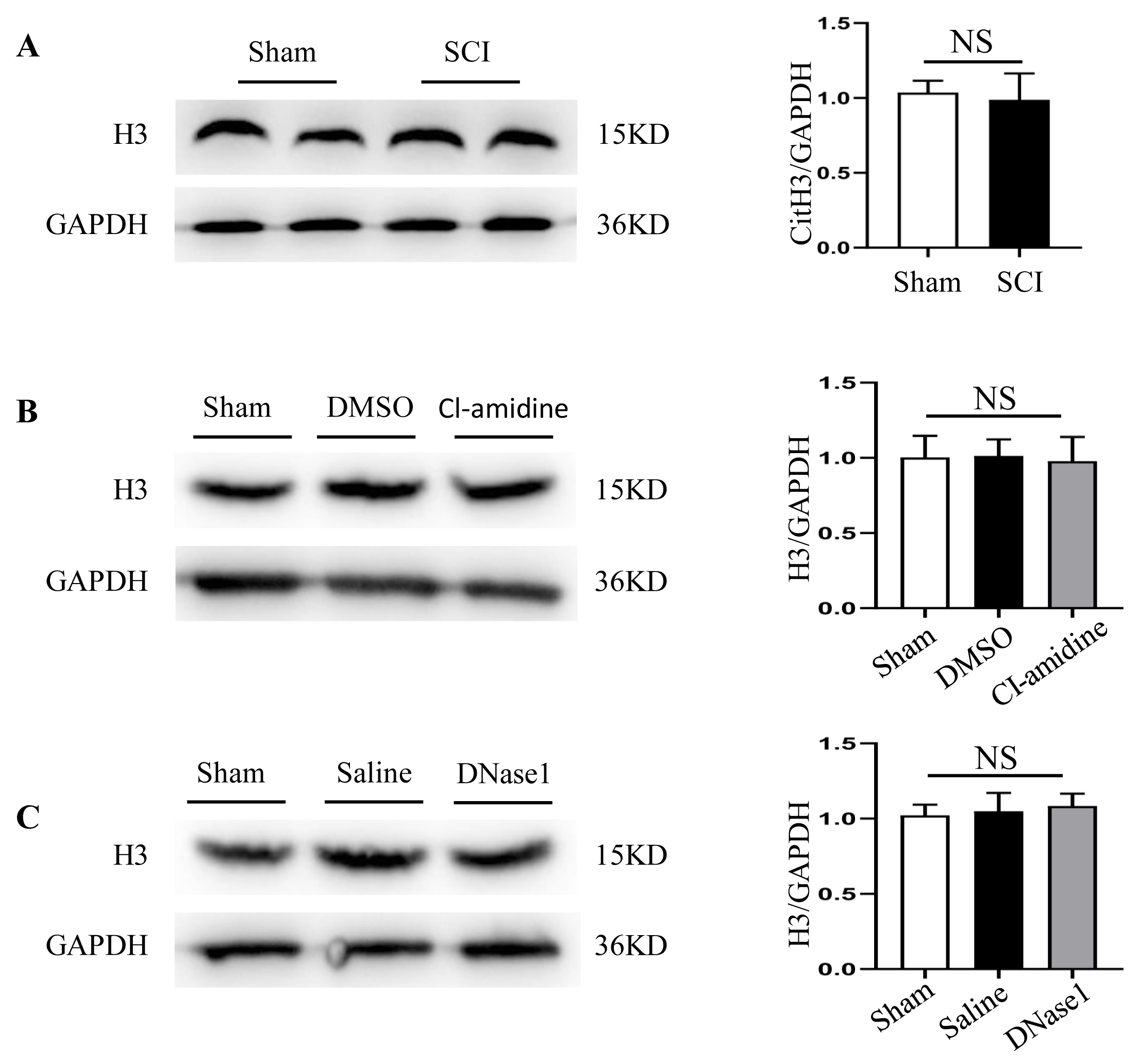

Supplement: Supplementary file 2 [file Image_2.tif]

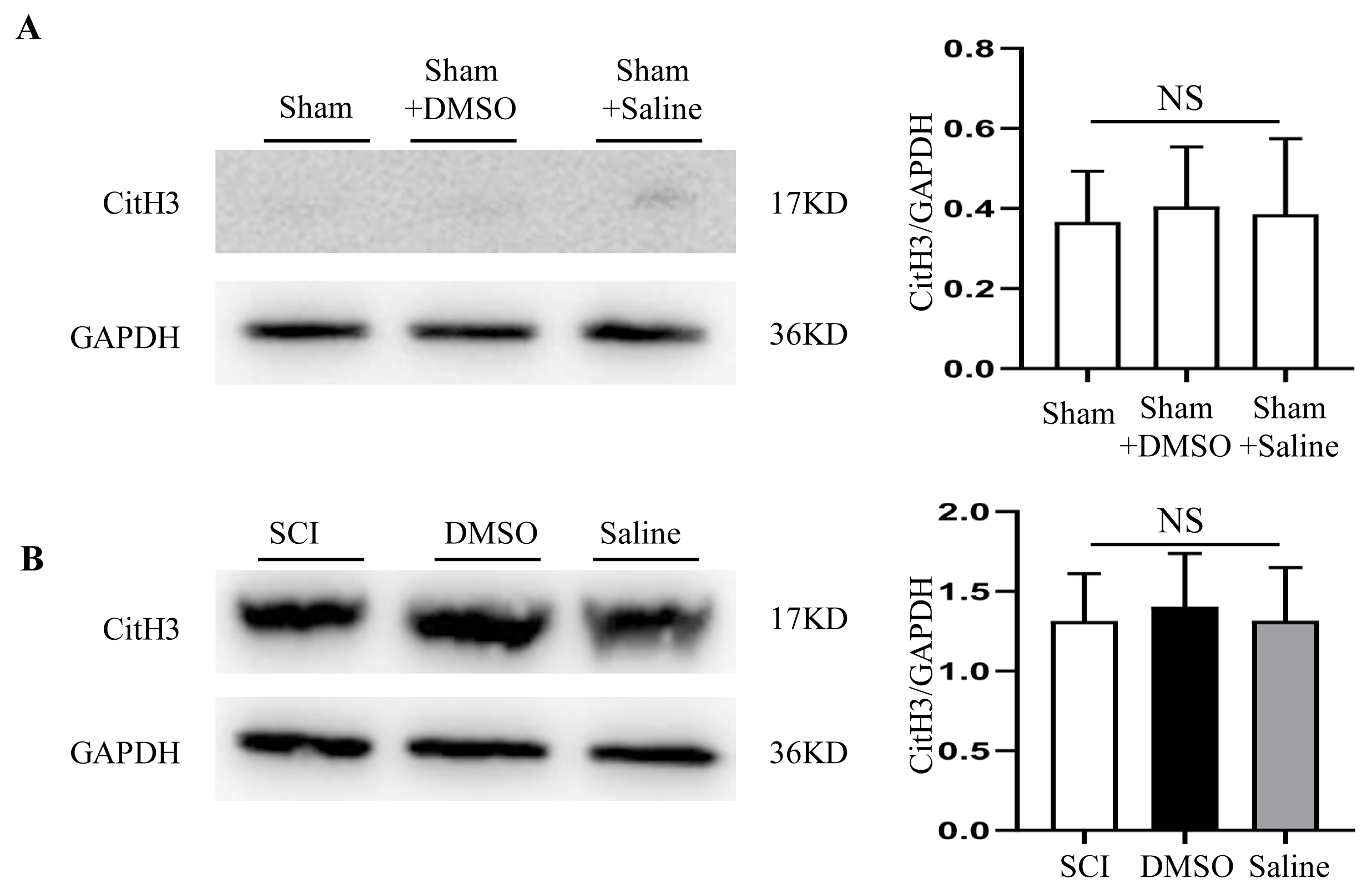

Supplement: Supplementary file 3 [file Image_3.tif]

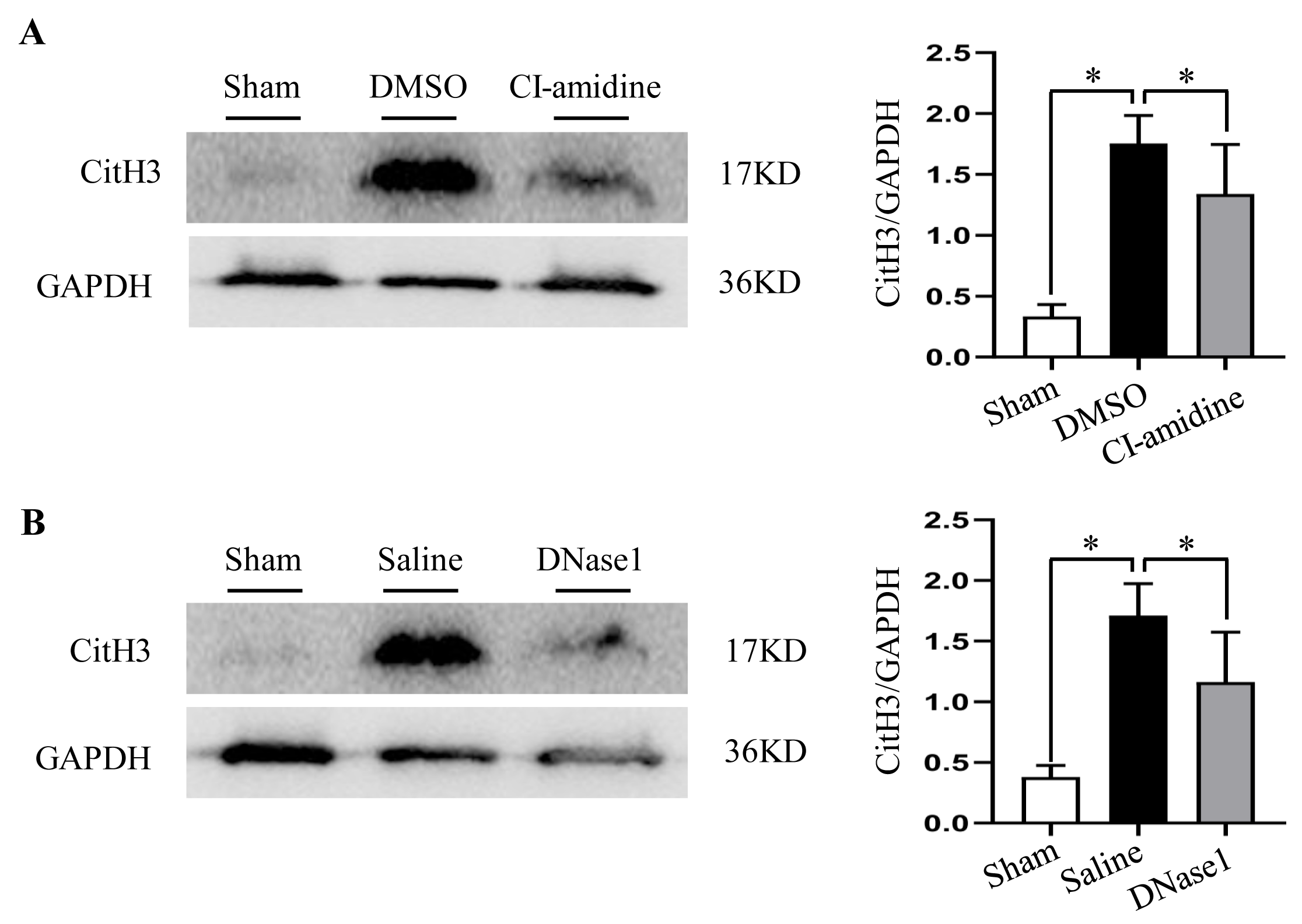

Supplement: Supplementary file 4 [file Image_4.tif]
